# Supplementary material for: Volume estimation models for avocado fruit
Source: PLoS One. 2022 Feb 3;17(2):e0263564. doi: 10.1371/journal.pone.0263564 (PMC8812970; doi:10.1371/journal.pone.0263564)
Supplement: S1 File — (PDF) [file pone.0263564.s001.pdf]

Supplementary Information

Volume estimation models for avocado fruit

Mulugeta Mokria[a*], Aster Gebrekirstos[b], Hadia Said[a], Kiros Hadgu[a], Niguse Hagazi[a], Workneh Dubale[c], Achim Bräuning[d]

[a]World Agroforestry (ICRAF), C/O ILRI Campus, Gurd Shola, P.O. Box 5689, Addis

Ababa, Ethiopia

[b]World Agroforestry (ICRAF), United Nations Avenue, P.O. Box 30677-00100, Nairobi, Kenya

[c]International Livestock Research Institute (ILRI), Addis Ababa, Ethiopia.

[d]Institute of Geography, Friedrich-Alexander-University Erlangen-Nuremberg, Wetterkreuz 15, 91058, Erlangen, Germany

[*]Corresponding author, E-mail: *m.mokria@cgiar.org; mgmokria@gmail.com;* ORCID: https://orcid.org/ **0000-0002-7812-8296**

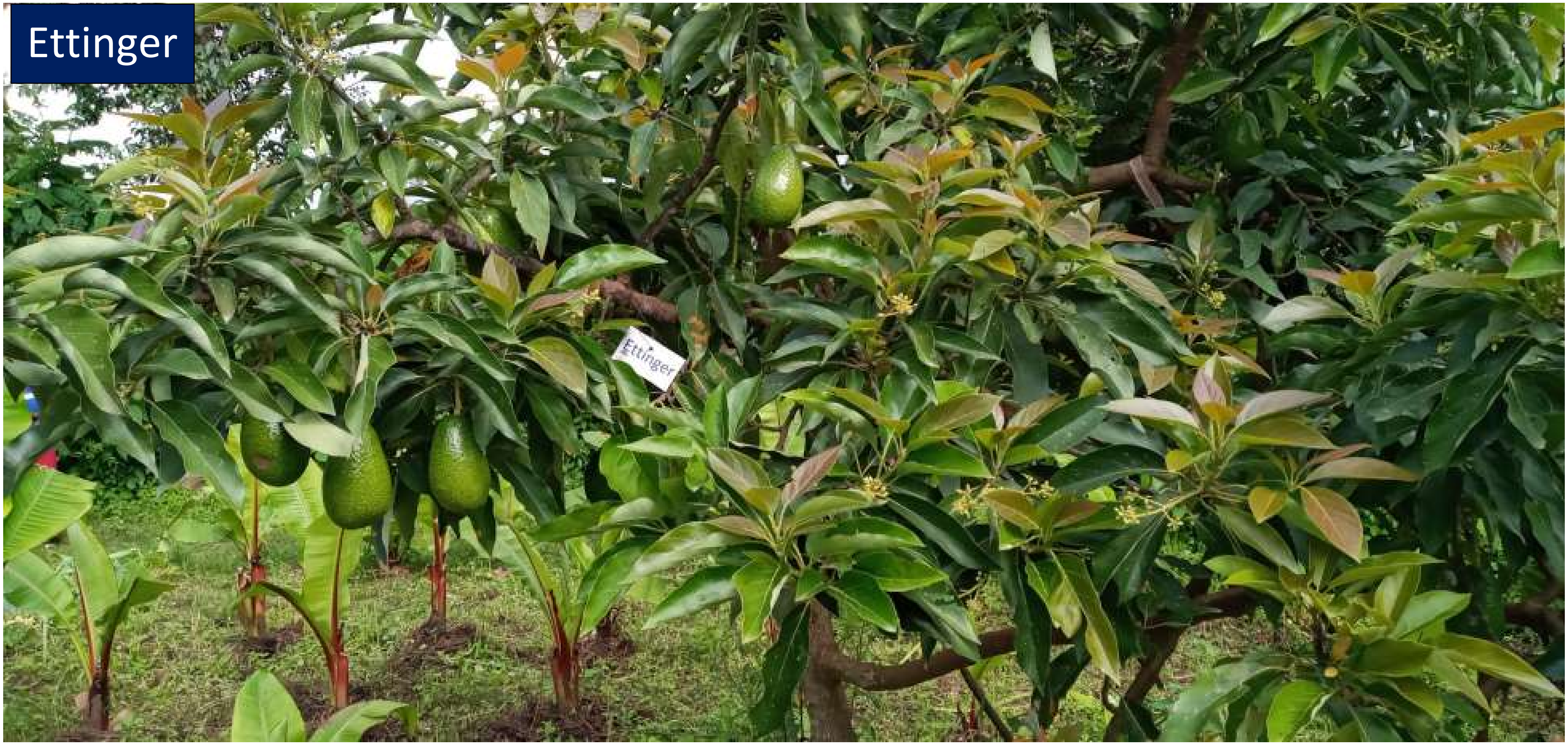

Source: ***Mulugeta Mokria***

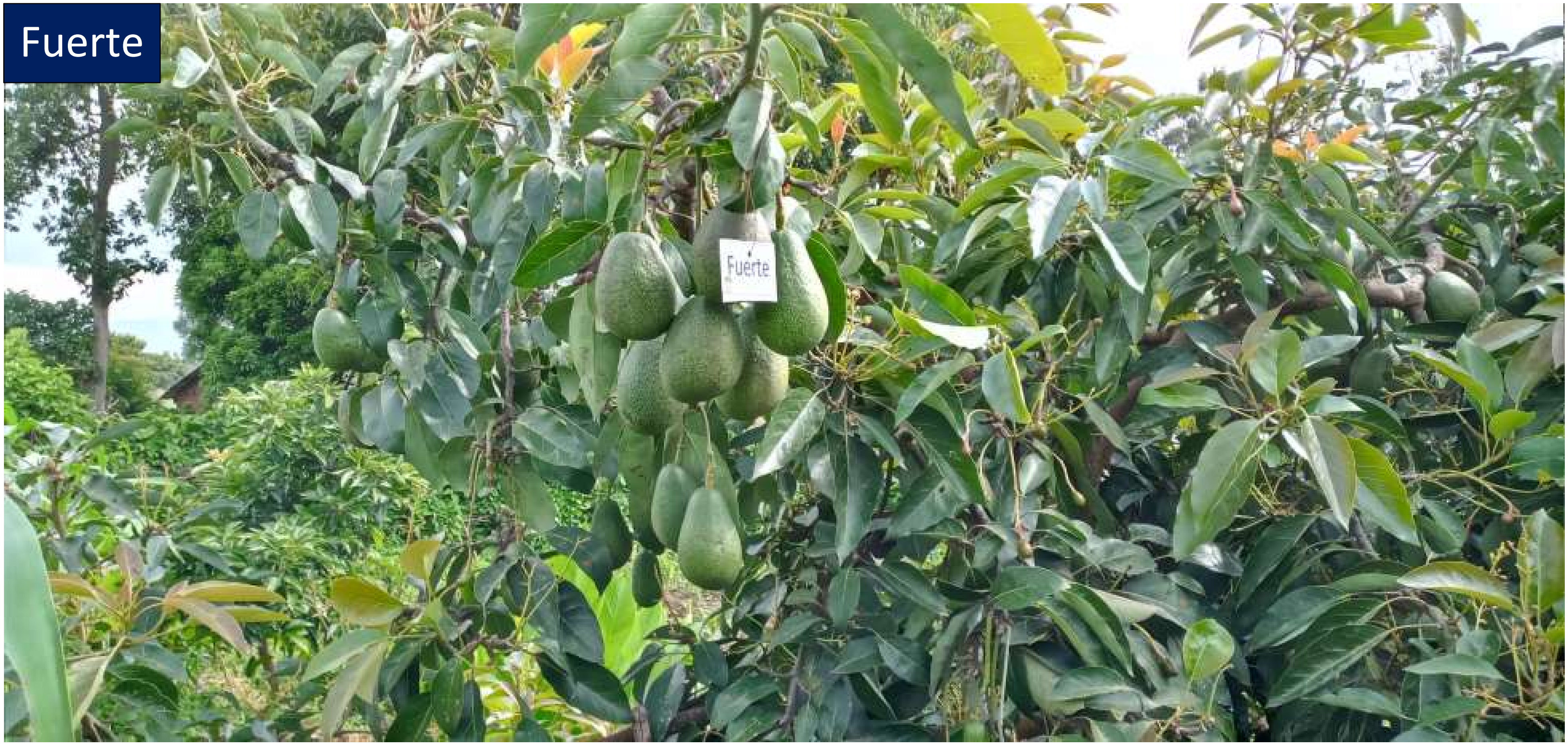

Source: ***Mulugeta Mokria***

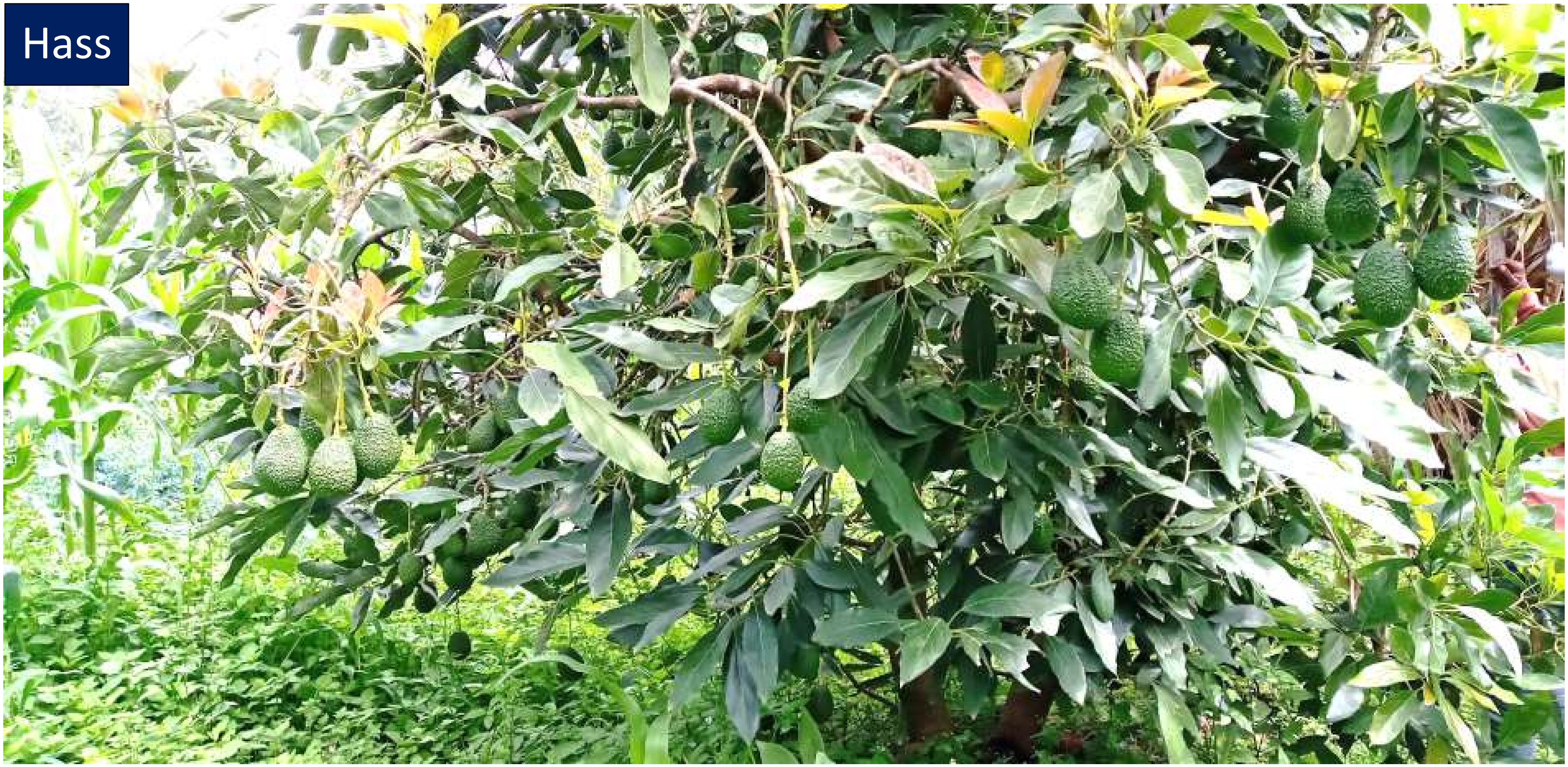

Source: ***Mulugeta Mokria***

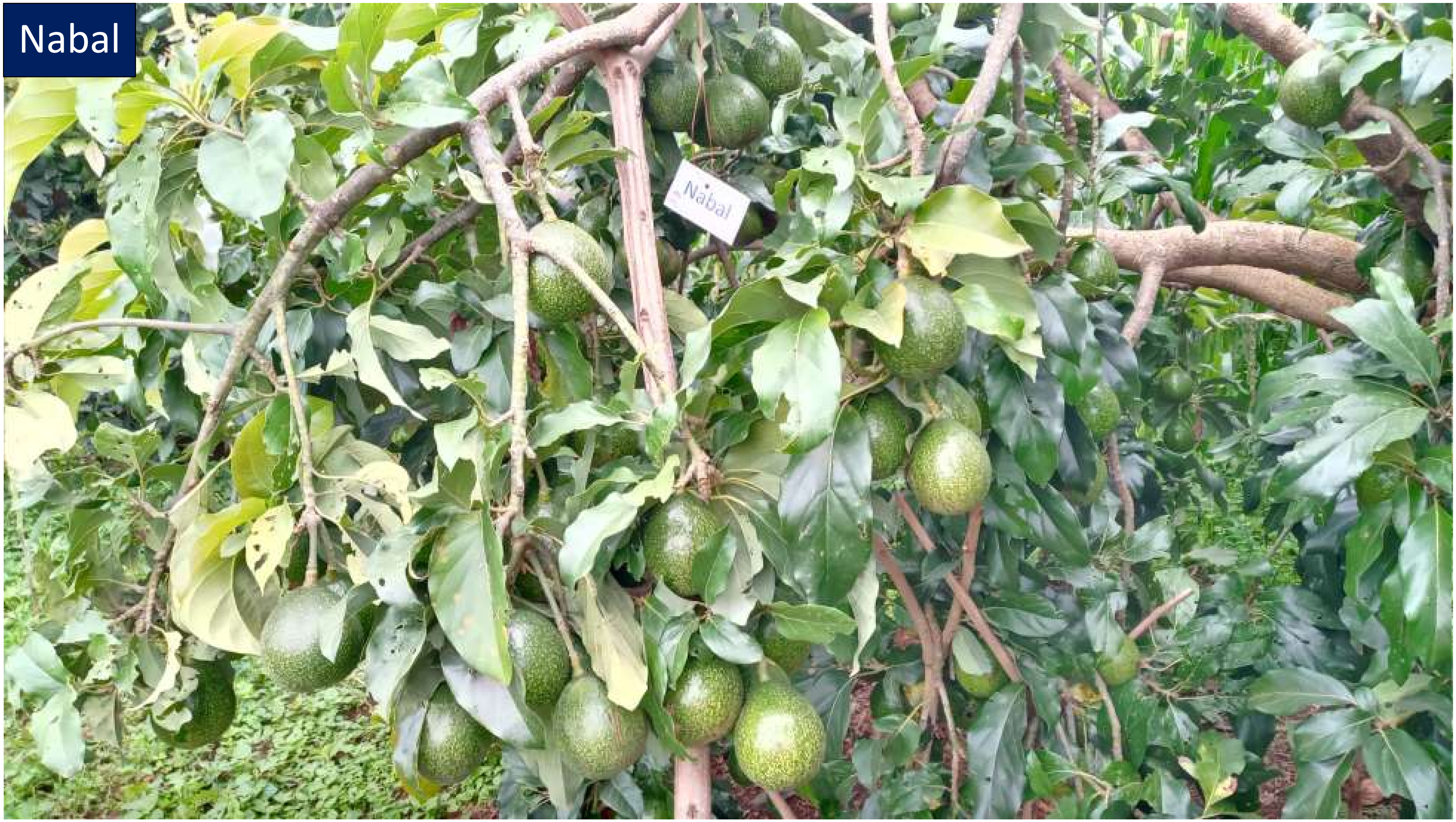

Source: *Mulugeta Mokria*

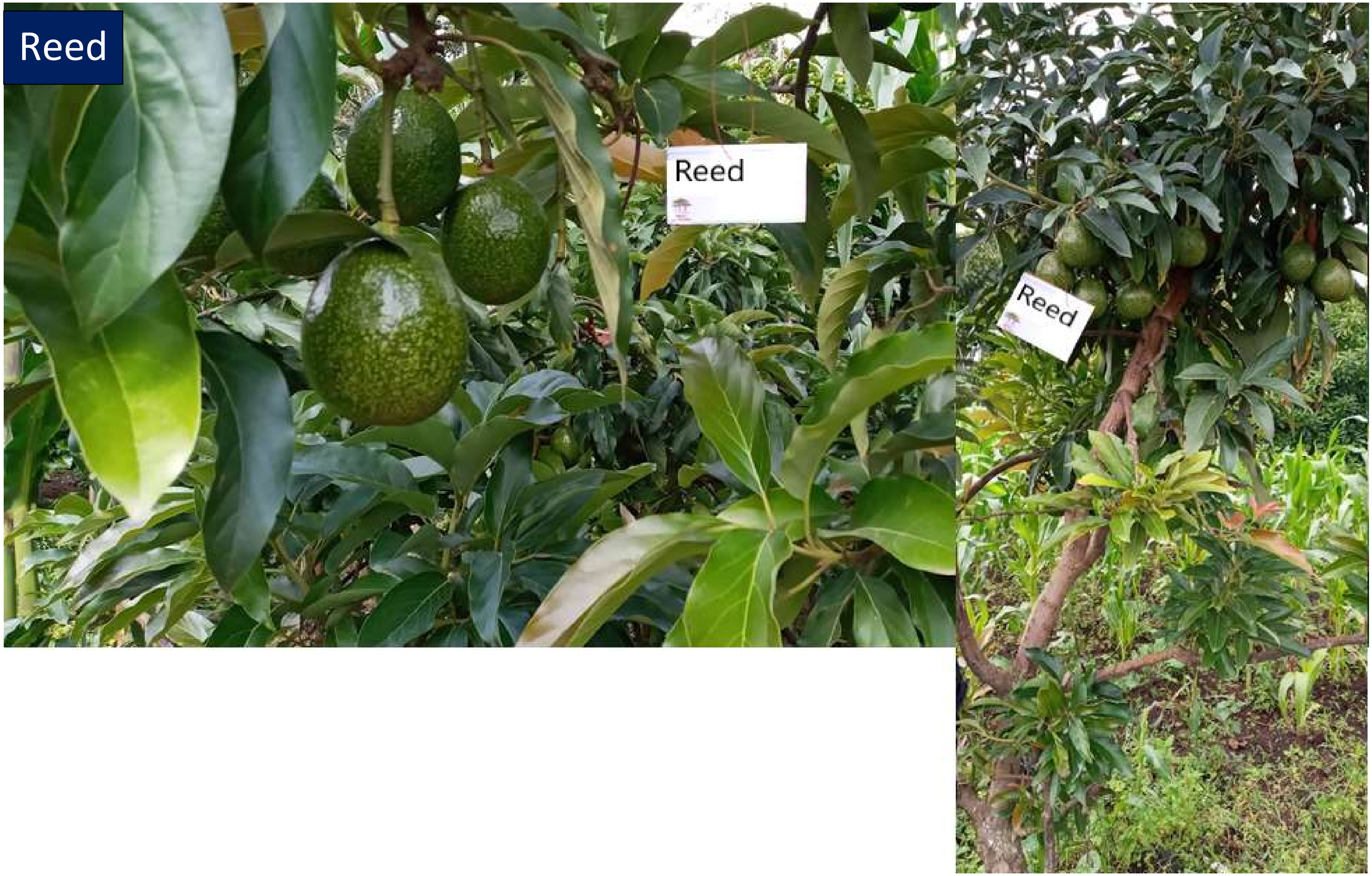

Source: *Mulugeta Mokria*
