# Supplementary material for: Volume estimation models for avocado fruit
Source: PLoS One. 2022 Feb 3;17(2):e0263564. doi: 10.1371/journal.pone.0263564 (PMC8812970; doi:10.1371/journal.pone.0263564)

PLOS ONE

Volume estimation models for tropical fruit

--Manuscript Draft--

**Manuscript Number:**	PONE-D-21-36255
**Article Type:**	Research Article
**Full Title:**	Volume estimation models for tropical fruit
**Short Title:**	Fruit Volume estimation models
**Corresponding Author:**	Mulugeta Mokria<br>ICRAF: World Agroforestry Centre<br>Addis Ababa, ETHIOPIA
**Keywords:**	Agroforestry; Avocado; Fruit; Volume Models; Ethiopia
**Abstract:**	Avocado (Persea americana Mill .) is a tropical tree, of the Lauraceae family, genus Persea. It is an important horticultural crop and proved to be a very profitable commercial crop for both local consumption and export. The physical characteristic of fruits are an important factor to determine the quality of fruit produced. On the other hand, estimation of fruit volume is time-consuming and impractical under field conditions. Thus, this study was conducted to devise cultivar-specific and generalized allometric models to analytically determine the non-destructive avocado fruit volume of five wildly distributed avocado verities. A significant relationship (P ≤ 0.01) was found between Fruit diameter, length, and volume of each cultivar. Our best models (VM2 – for cultivar specific, and VM7-generalized model) has passed all the rigorous cross-validation and performance statistics tests and explained 94%, 92%, 87%, 93%, 94% and 93% of the variations in fruit volume of Ettinger, Fuerte, Hass, Nabal, Reed30, and Multiple cultivars, respectively. Our finding revealed that in the situations where measurements of volume would be inconvenient, or time consuming, a reliable volume and yield estimation can be obtained using site- and cultivar-specific allometric equations. Allometric models could also play a significant role in improving data availability on avocado fruit physical appearance which is critical to assess the quality and taste of fresh product influencing the purchase decision of customers. Moreover, such information can also be used as a ripeness index to predict optimum harvest time important for planned marketing. More importantly, the models might assist horticulturists, agronomists, and physiologists to conduct further study on avocado production and productivity through agroforestry landuse system across Ethiopia
**Order of Authors:**	Mulugeta Mokria
	Aster Gebrekirstos, PhD
	Hadia Said
	Kiros Hadgu, PhD
	Niguse Hagazi
	Workneh Dubale
	Achim Bräuning
**Additional Information:**	
**Question**	**Response**
**Financial Disclosure**<br><br>Enter a financial disclosure statement that describes the sources of funding for the work included in this submission. Review the submission guidelines for detailed requirements. View published research articles from *PLOS ONE* for specific	This study was conducted with the financial support of the Africa Rising Project- a program financed by the United States Agency for International Development (USAID) as part of the United States Government’s Feed the Future Initiative.

Powered by Editorial Manager® and ProduXion Manager® from Aries Systems Corporation

examples.<br><br>This statement is required for submission and **will appear in the published article** if the submission is accepted. Please make sure it is accurate.<br><br>**Unfunded studies**<br>Enter: *The author(s) received no specific funding for this work.*<br><br>**Funded studies**<br>Enter a statement with the following details:<br>• Initials of the authors who received each award<br>• Grant numbers awarded to each author<br>• The full name of each funder<br>• URL of each funder website<br>• Did the sponsors or funders play any role in the study design, data collection and analysis, decision to publish, or preparation of the manuscript?<br>• **NO** - Include this sentence at the end of your statement: *The funders had no role in study design, data collection and analysis, decision to publish, or preparation of the manuscript.*<br>• **YES** - Specify the role(s) played.<br><br>* typeset	
**Competing Interests**<br><br>Use the instructions below to enter a competing interest statement for this submission. On behalf of all authors, disclose any competing interests that could be perceived to bias this work—acknowledging all financial support and any other relevant financial or non-financial competing interests.<br><br>This statement is required for submission and **will appear in the published article** if the submission is accepted. Please make sure it is accurate and that any funding sources listed in your Funding Information later in the submission form are also declared in your Financial Disclosure statement.	The authors have declared that no competing interests exist.

View published research articles from *PLOS ONE* for specific examples.<br><br>**NO authors have competing interests**<br><br>Enter: *The authors have declared that no competing interests exist.*<br><br>**Authors with competing interests**<br><br>Enter competing interest details beginning with this statement:<br><br>*I have read the journal's policy and the authors of this manuscript have the following competing interests: [insert competing interests here]*<br><br>* typeset	
**Ethics Statement**<br><br>Enter an ethics statement for this submission. This statement is required if the study involved:<br><br>• Human participants<br>• Human specimens or tissue<br>• Vertebrate animals or cephalopods<br>• Vertebrate embryos or tissues<br>• Field research<br><br>Write "N/A" if the submission does not require an ethics statement.<br><br>General guidance is provided below. Consult the submission guidelines for detailed instructions. **Make sure that all information entered here is included in the Methods section of the manuscript.**	N/A

Powered by Editorial Manager® and ProduXion Manager® from Aries Systems Corporation

**Format for specific study types**<br><br>**Human Subject Research (involving human participants and/or tissue)**<br>• Give the name of the institutional review board or ethics committee that approved the study<br>• Include the approval number and/or a statement indicating approval of this research<br>• Indicate the form of consent obtained (written/oral) or the reason that consent was not obtained (e.g. the data were analyzed anonymously)<br><br>**Animal Research (involving vertebrate animals, embryos or tissues)**<br>• Provide the name of the Institutional Animal Care and Use Committee (IACUC) or other relevant ethics board that reviewed the study protocol, and indicate whether they approved this research or granted a formal waiver of ethical approval<br>• Include an approval number if one was obtained<br>• If the study involved *non-human primates*, add *additional details* about animal welfare and steps taken to ameliorate suffering<br>• If anesthesia, euthanasia, or any kind of animal sacrifice is part of the study, include briefly which substances and/or methods were applied<br><br>**Field Research**<br><br>Include the following details if this study involves the collection of plant, animal, or other materials from a natural setting:<br>• Field permit number<br>• Name of the institution or relevant body that granted permission	
**Data Availability**<br><br>Authors are required to make all data underlying the findings described fully available, without restriction, and from the time of publication. PLOS allows rare exceptions to address legal and ethical concerns. See the PLOS Data Policy and FAQ for detailed information.	Yes - all data are fully available without restriction

Powered by Editorial Manager® and ProduXion Manager® from Aries Systems Corporation

A Data Availability Statement describing where the data can be found is required at submission. Your answers to this question constitute the Data Availability Statement and **will be published in the article**, if accepted.<br><br>**Important:** Stating 'data available on request from the author' is not sufficient. If your data are only available upon request, select 'No' for the first question and explain your exceptional situation in the text box.<br><br>Do the authors confirm that all data underlying the findings described in their manuscript are fully available without restriction?	
**Describe where the data may be found in full sentences. If you are copying our sample text, replace any instances of XXX with the appropriate details.**<br><br>• If the data are **held or will be held in a public repository**, include URLs, accession numbers or DOIs. If this information will only be available after acceptance, indicate this by ticking the box below. For example: *All XXX files are available from the XXX database (accession number(s) XXX, XXX.).*<br>• If the data are all contained **within the manuscript and/or Supporting Information files**, enter the following: *All relevant data are within the manuscript and its Supporting Information files.*<br>• If neither of these applies but you are able to provide **details of access elsewhere**, with or without limitations, please do so. For example:<br><br>*Data cannot be shared publicly because of [XXX]. Data are available from the XXX Institutional Data Access / Ethics Committee (contact via XXX) for researchers who meet the criteria for access to confidential data.*<br><br>*The data underlying the results presented in the study are available from (include the name of the third party*	All relevant data are within the manuscript and its Supporting Information files.

and contact information or URL).<br>• This text is appropriate if the data are owned by a third party and authors do not have permission to share the data.<br><br>* typeset	
Additional data availability information:	

Manuscript
Click here to access/download;Manuscript;Mokria et al_2021_Manuscript_PLOS.doc

Volume estimation models for tropical fruit

Mulugeta Mokria[a*], Aster Gebrekirstos[b], Hadia Said[a], Kiros Hadgu[a], Niguse Hagazi[a], Workneh Dubale[c], Achim Bräuning[d]

[a]World Agroforestry (ICRAF), C/O ILRI Campus, Gurd Shola, P.O. Box 5689, Addis Ababa, Ethiopia

[b]World Agroforestry (ICRAF), United Nations Avenue, P.O. Box 30677-00100, Nairobi, Kenya

[c]International Livestock Research Institute (ILRI), Addis Ababa, Ethiopia.

[d]Institute of Geography, Friedrich-Alexander-University Erlangen-Nuremberg, Wetterkreuz 15, 91058, Erlangen, Germany

[*]Corresponding author, E-mail: *m.mokria@cgiar.org; mgmokria@gmail.com;* ORCID: https://orcid.org/ **0000-0002-7812-8296**

Abstract

Avocado (*Persea americana Mill.*) is a tropical tree, of the Lauraceae family, genus Persea. It is an important horticultural crop and proved to be a very profitable commercial crop for both local consumption and export. The physical ~~characteristic~~ of fruits are ~~an~~ important factor to determine the quality of fruit produced. On the other hand, estimation of fruit volume is time-consuming and impractical under field conditions. Thus, this study was conducted to devise cultivar-specific and generalized allometric models to analytically determine the non-destructive avocado fruit volume of five wildly distributed avocado verities. A significant relationship ($P \leq 0.01$) was

found between Fruit diameter, length, and volume of each cultivar. Our best models (VM2 – for cultivar specific, and VM7-generalized model) has passed all the rigorous cross-validation and performance statistics tests and explained 94%, 92%, 87%, 93%, 94% and 93% of the variations in fruit volume of Ettinger, Fuerte, Hass, Nabal, Reed30, and Multiple cultivars, respectively. Our finding revealed that in the situations where measurements of volume would be inconvenient, or time consuming, a reliable volume and yield estimation can be obtained using site- and cultivar-specific allometric equations. Allometric models could also play a significant role in improving data availability on avocado fruit physical appearance which is critical to assess the quality and taste of fresh product influencing the purchase decision of customers. Moreover, such information can also be used as a ripeness index to predict optimum harvest time important for planned marketing. More importantly, the models might assist horticulturists, agronomists, and physiologists to conduct further study on avocado production and productivity through agroforestry landuse system across Ethiopia.

Key words: Agroforestry, Avocado, Fruit, Volume Models, Ethiopia

1. Introduction

Avocados are an evergreen subtropical species and the most economically important species of the Lauraceae family. It is grown commercially in America, Africa, Europe, Asia and Oceania. In 2019, the estimated world's total avocado production was about 7.2 million tonnes and covering the total production areas of 726, 660 hectares (1). The major avocado growing countries are Mexico, USA, Colombia, Indonesia, Chile, the Dominican Republic, Kenya and South Africa (1). In Africa, Kenya and South Africa are leading in the production and export of avocado to the global market (2). In Ethiopia, it was first introduced around 1938 in the eastern and southern parts of the country and it is now being widely

distributed throughout the country, mainly used for household consumption and local market (3–5). Currently, Ethiopia is one of the top five avocado producing country in sub-Saharan Africa (SSA) and the 20th in the world (1). Avocado is recognized that besides being a source of energy and vitamins, it also provides specific non-nutritive physiological benefits that may enhance health (6), thus, it can be considered as a "functional food" (7). It is one of the top important commercial crop to be traded at a global scale (8,9), and becoming one of the most promising fruit crops for both food and nutrition security and earning considerable amount of financial return from export and domestic market (10–12). Due to government initiatives in promoting investment in horticulture sector as well as combating climate change through land diversification and agroforestry practices, the plantation and the production of avocado is considerably increasing over the last few years across different parts of Ethiopia. Despite the expansion of avocado tree plantation, the physical characteristic of avocado fruits and productivity of small scall avocado farming are not well studied in Ethiopia. On the other hand, information of fruit size are critical factors to determine the quality of the avocados and has been used to describe the fruit's growth curve, predict yield, and conduct physiological studies (13). More importantly, on-tree and non-destructive volume estimates can be used as a ripeness index to predict maturity and optimum harvest time, and can inform packing material (tray insert) purchasing and marketing arrangements (14). For physiological studies, measurement of the size of individual fruit over time allows to monitor fruit expansion rate and its response to physiological disorders and agronomic conditions (14,15). Moreover, in the context of postharvest operations, fruit size determination is important for several reasons, such as to determine packing material, fruit classified into batches of uniform size, assign market and price differentials of large and small produce, to match consumer preferences (16). Thus, the availability of reliable fruit size information is critical in horticultural crop processing and marketing. Fruit volume is also a good measure of size, but direct measurement of fruit

volume using water displacement approach is ~~expensive and~~ time-consuming as well as impractical under field conditions. On the other hand, length and width measurements of avocado fruit are quick and easy in the field or indoors and can be used to numerically represent fruit volume and weight. Therefore, this study was conducted to: (a) determine fruit volume of five avocado ~~verities,~~ (b) determine which biometric parameter of the avocado fruit best correlates with volume; (c) derive various cultivar-specific and mixed-cultivar allometric equations to predict fruit volume and (d) to evaluate the predictive performances of the equations and to identify the best allometric equation for the study region.

2. Materials and methods

2.1 Study area and climate characterises.

The study was conducted in the Upper Gana (7° 34' 24'' N, 37° 46' 4''E) and Jewe (7° 30' 35'' N, 37° 47' 1'') Kebeles of Limu district, situated in Hadiya zone, in the Ethiopian Southern Nations, Nationalities, and Peoples' Region (SNNPR) (17) (Fig. 1). The study area is located at 223 km South of Addis Ababa. The altitudinal ranges of Jewe and upper-Gana Kebles were between 2000 and 2400m ~~a.s.l~~ (Fig. 1). Based on the data from CSA ~~2007,~~ the district has an estimated total population of 153,783 and 93% of the people are living in the rural areas and practicing subsistence farming depending on rain fed production system (18). The average farm size per family head was estimated to be 0.5 ha (17).

In the study area, the annual rainfall ranges between 1300 and 1400 mm with a bi-modal rainfall seasonality , occurring from February to April and from June to September (17). The average annual minimum and maximum temperatures were 18 °C and 23 °C (17). The typical landuse system of the district is characterized by Agroforestry (i.e., mixed crop-tree-livestock production). (19). The district has a favourable climate and agroecological condition for multi-strata agroforestry and home garden intensive farming system.

Fig. 1. The location of the study area in Hadiya zone, Ethiopia

2.2. Tree selection for sample fruit collection

In this study, five different avocado cultivars including Ettinger (E), Fuerte (F), Hass (H), Nabal (N), and Reed30 (R) were considered. From each cultivar, 30 ~~(thirty)~~ avocado trees, ~~which~~ representing 30 small scale farmlands, were selected for fruit sample collection. Fruit ~~sample~~ were collected ~~following the~~ considering each radii of the crown (20–23). A total of 360 ~~sample~~ ~~fruits~~ were randomly harvested from each cultivar (i.e., three fruits from each radius or 12 sample fruits per tree = 12 fruit ~~*~~30 trees = 360 fruits). Then, ~~Fruit~~ length (FL in mm), diameter (FD in mm) and weight (FW in g) were measured using digital calliper and scale, respectively. For fruit volume measurement, the collected sample fruits were first classified ~~in to~~ three size ~~class~~ (small, medium, and large). Then, ~~fifteen (15)~~ fruits were randomly selected from each size class (total = 45 fruits from each cultivar). For each fruit, actual fruit volume (AFV) was measured using water displacement method (13,24), in the Biotechnology Laboratory of Wachemo University (WCU), Hossana, Ethiopia.

2.3. Model development, performance evaluation and cross-validation test

Cultivar-specific and mixed-cultivar generalized avocado fruit volume (FV) estimation models were developed using linear and non-linear regression equations based on either fruit diameter, fruit length alone or both fruit length and diameter at the same time as independent variables. Moreover, using two predictors (i.e., length and diameter) may introduce potential problems of co-linearity, resulting in poor precision in the estimates of the corresponding regression coefficients (25). Thus, for detecting the variance inflation factor (VIF = 1/(1-r^2) and the tolerance values (T = 1/VIF) were calculated (26). Where r is the correlation coefficient between length and diameter of fruit. VIF value exceeding 10 or if T value was smaller than 0.10 then co-

linearity may have a considerable impact on the prediction of the parameters, and consequently one of those should be excluded from the model (27,28).

Model performance was checked using various goodness-of-fit statistics, such as the Coefficient of Determination (R^2), Standard Error of Estimate (SEE), Index of Agreement (D), Mean Absolute Bias (MAB), Percent Bias (PBIAS), Root Mean Square Error (RMSE), Prediction Residuals Sum of Squares (PRESS), Reduction of error (RE), and Coefficient efficiency (CE), (29–31). The estimation models with higher R^2 may sometimes also have unstable parameters estimate. Thus, we further calculated Percent Relative Standard Error (PRSE) of the coefficients and Weighted Akaike information criterion (AICiw) to check the stability of model parameter estimates (25). Outlier and influential diagnostic test statistics, including Cook's distance, Leverage point, Studentized Residuals and DFFITS were analysed to examine the accuracy of model fit (25,32). Finally, Models were evaluated and ranked based on all goodness-of-fit statistics, outlier, and influential diagnostic statistics (31).

To validate the best fitting equation for volume estimation, model cross-validation was conducted following a split-sample approach in which 45 measured fruit sample were partitioned into two sets, 33 for ‘‘training’’ (i. e., to develop the equations) and the remaining 12 fruit samples for ‘‘testing’’ the equations. The partitioning was performed according to the following procedure. From each size class (small, medium, and large) of the samples, four samples were randomly selected to form the ‘‘test’’ dataset and the remaining sample fruits were used to form the ‘‘training’’ dataset. The goodness-of-fit statistics and equation coefficients of the ‘‘training’’ equations were compared with those derived using the full dataset, and the estimated and measured volume of ‘‘test’’ sample fruit compared (31,33,34). Finally, the full dataset was used to build fruit volume estimation models.

2.4. Statistical analysis

Pearson correlation tests were conducted between actual Fruit volume (AFV)-fruit diameter (FD) and fruit length (FL) to be able to identify which fruit biometric variables were most strongly correlated with fruit volume (FV). A correlation analysis was also conducted between independent variables (i.e., FL vs FD). The differences among avocado varieties in AFV was assessed using one-way analysis of variance and the significance of differences were tested using the least significant difference test (LSD) with $P < 0.05$ (31).

3. Result and discussion

3.1. The relationship of physical characteristics of avocado fruits

The physical characteristics of sample avocado fruits and their statistical attributes are presented in Table 1. The actual fruit volume (FV) ranged from 125 – 480 cm^3, fruit weights (FW) ranged from 129 – 595 g, fruit length (FL) ranged from 64.5- 129.9 mm, and fruit diameter (FD) ranged from 53.8 – 99.8 mm (Table 1). These findings are in line with values in other reports (35–37). Information on fruit physical appearance is important for the customer who is used to assess the quality and taste of fresh product influencing the purchase decision (14).

The relationship between fruit volume and other pomological traits (i.e., FL, FD, FV) were significant ($P < 0.01$) (Fig. 2) and that is in agreement with other studies that have showed strong relationships between different pomological traits of avocado fruit (36). Moreover, the correlation between FD and FV were consistently stronger compared to the relationship between FL and FV (Fig. 2), indicating the FD is a very good predictor of fruit volume and highlighting those changes in diameter, therefore, affects the fruit volume more than does a change in fruit length. More importantly, knowing the relationship between fruit physical characteristics is critical in the horticultural sector because these pomological traits are sometime used as fruit

maturity index (36,38,39). Several indices have been used to determine avocado fruit maturity, hence ~~there is~~ no single factor can be considered the most important; however, it can be ~~said~~ that from a postharvest standpoint, quality begins at harvest with physiological maturity. This indicates that understanding the stage of physiological maturity is critical for the development of a successful avocado fresh fruit industry while assuring the quality to the consumer (40).

Fig. 2. Fruit volume as a function of fruit length, diameter, and regression of fruit diameter as a function of fruit length.

Table 1. Summary of volume, and fruit size characteristics of five avocado cultivars

	Fruit Length (mm)		Fruit Diameter (mm)		Fruit wight (g)		Fruit Volume (~~cm3~~)	
SPP name	Mean [SE]	Range	Mean [SE]	Range	Mean [SE]	Range	Mean [SE]	Range
Ettingenr	108.9 [±1.5]	91.9 -129.9	69.1 [±0.9]	57.1-84.2	253 [±8.8]	162-375	274.1 [±10.1]	145-410
Fuerte	106.2 [±1.4]	87.7 -124.6	68.4 [±0.8]	57.4-79.6	248.8 [±8.2]	146-348	252.0 [±7.4]	150-350
Hass	90.3 [±1.4]	72.8 -113.9	64.7 [±0.8]	53.8 -76	192.6 [±7.0]	116-310	181.6 [±5.8]	110-300
Nabal	92.6 [±1.5]	70.1 -118.7	82.6 [±1.2]	65.8-99.8	331.9 [±13.8]	160-595	324.7 [±11.3]	180-480
Reed_30	81.3 [±1.3]	64.5 -98.1	74.1 [±1.1]	60.8-89.2	242.3 [±10.3]	129-426	247.1 [±10.4]	125-420

3.2. Allometric equations and model cross-validation

As a preliminary step to model calibration, the degree of collinearity among fruit length and diameter was analysed. The VIF was ranged from 1.1 to 4.6 and T values ranged from 0.21 to 0.95, depending on cultivar type, respectively (Supplementary information (S). Hence, for all selected genotypes, VIF was < 10 and T was > 0.10, showing that the co-linearity between predictors (fruit length and diameter) is negligible, thus both predictors (FL, FD) were considered during model formation (27,28) (S).

Cultivar specific and mixed cultivar generalized volume model was developed to approximate the shape of an avocado fruits using either fruit length (FL) or width (FD) separately and using both predictors at a time. We found that the predictive performance of tested model form were varied within cultivar (Table 2, S). This might be attributed to differences in the equation forms and predictors included in the models (31). Allometric model performance analysis and cross-

validation test results showed that the Linear Regression Model (MV2) which includes only FD as predictor was ranked the best model given the set of nine (9) candidate model forms for all cultivar-specific models, while the Multiple Linear Regression Model (VM7) was the best for generalized mixed cultivar model (Table 2, S).

Our best model explained 94%, 92%, 87%, 93%, 94% and 93% of fruit volume in Ettinger, Fuerte, Hass, Nabal, Reed30 and mixed-avocado cultivar model, respectively (Fig. 3, Table 2, in Supporting Information for more details). The three best performing models for each avocado cultivar and mixed-cultivar are shown in Table 2, where the influence of coefficients was significant ($P < 0.01$) (Table 2). Our best model (VM2 for cultivar specific) and VM7 – for mixed cultivar models have passed all the rigorous verification and cross-validation statistical test and produced the lowest average relative error (PBIAS%), implying that fruit diameter is reliable predictors of cultivar specific fruit volume, while using both FL and FD might increase the predictive performances of generalized allometric models (S). Moreover, the performance of our best models (VM2, and VM7) to make an accurate prediction is not an artifact of over fitting, because the parameter values were stable across the subset of the cross-validations "training data set" and full data set (S). Moreover, the volume of the twelve ''test'' fruit volume estimated with the cross-validation equations (i.e., training dataset) differed little from the values estimated with equations produced with the full dataset (Fig. 3, S). The deviations (PBIAS%) in the volume estimates between the two sets of equations were less than 1% for all and mixed cultivar equations (Fig. 3). Moreover, the PRSE value of < 20% and outliers and influential points of less than 10% of the total observation as well as higher positive value of CE, and RE provides evidence that the parameter estimates were reliable in the selected best models (25,41–43) (S). Thus, VM2 (i.e., cultivar specific model) and VM7 (i.e., generalized model) are reliable to

determine the fruit volume based on their easily measurable fruit pomological traits (i.e., FL or/and FD). Furthermore, measuring fruit length and diameter are easy in the field, thus site-and cultivar specific allometric model would enable researchers to make non-destructive or repeated measurements on the same fruits. Our allometric models could provide accurate estimate of avocado fruit volume and may reduce required time and financial resources while using common method of volume measurements like water displacement, gas displacement and expensive instruments, e.g., image processing software or machine vision techniques. In line with this, a review literature showed that there are different non-distractive volume estimation models developed for different type of fruits ~~from easily measurable parameters~~, such as ~~for~~ pepper (13,15), ~~for~~ Babassu (*Attalea speciosa)* fruit (44), Karanda (*Carissa carandas*) fruit (27), ~~for Apple~~ fruit (45). Our ~~finding~~ confirmed that non-destructive allometric models based on easily measurable morphometric dimensions can be more accurate and practical in field conditions than destructive methods used in traditional growth curves (16).

Fig. 3. Relationships between the ~~Fruit~~ volume each cultivar and fruit diameter (left panel) and corresponding plots of residuals (modelled minus measured fruit volume values) (right). Fig.3a-F1/F2 refers Ettinger, Fuerte, Hass, Nabal, Reed30 and Mixed cultivar.

Table 2. Equations and goodness-of-fit performance statistics for estimating avocado fruit volume of five different ~~cultivar~~ and multiple cultivars grown in Limo district, Hadiya~~,~~ zone.

Model forms	Model forms	Model parameter a	b	c	Performance statistics R2	SEE	PRESS	RMSE	PBIAS	MAB	Di	RE	CE	AICi	Δi(AIC)	Wi(AIC)	PRSE a	b	c	Rank
Ettinger																				
M2	a*FD + b	**10.4484**	**-447.701**		0.94	17.53	13210.88	17.13	0.00	12.20	0.98	1.00	0.94	259.70	11.31	0.00	4.0	-6.5		**1**
M4	a*FD^2	**0.0575**			0.88	24.19	25752.29	23.92	-0.94	18.94	0.96	0.99	0.88	287.73	39.34	0.00	1.2			**2**
M9	a*(FL*FD)^2	**4.53E-06**			0.74	35.17	54412.87	34.77	2.71	28.37	0.95	0.98	0.74	321.40	73.01	0.00	1.9			**3**
Fuerte																				
M2	a*FD + b	**8.369**	**-320.756**		0.92	14.81	9425.88	14.47	0.00	10.93	0.98	1.00	0.92	244.50	0.22	0.46	4.6	-8.3		**1**
M4	a*FD^2	**0.0536**			0.89	16.33	11733.34	16.15	-0.31	12.71	0.97	1.00	0.89	252.36	8.07	0.01	0.9			2
M7	a+ b*FL + c*FD	**-333.124**	0.5107	**7.757**	0.92	14.62	8973.05	14.12	0.00	10.62	0.98	1.00	0.92	244.29	0.00	0.52	-8.3	**68.5**	7.3	3
Hass																				
M2	a*FD + b	**6.5239**	**-240.661**		0.87	14.33	8832.76	14.01	0.00	9.00	0.96	0.99	0.87	306.82	69.78	0.00	5.8	-10.3		**1**
M4	a*FD^2	**0.0432**			0.86	14.65	9438.43	14.48	-0.41	10.30	0.96	0.99	0.86	237.04	0.00	0.71	1.2			2
M7	a+ b*FL + c*FD	**-252.658**	0.5479	**5.9447**	0.88	13.93	8149.19	13.46	0.00	9.03	0.97	0.99	0.88	239.96	2.91	0.17	-9.9	**53.3**	8.1	3
Nabal																				
M2	a*FD + b	**9.314**	**-444.277**		0.93	20.20	17549.35	19.75	0.00	14.44	0.98	1.00	0.93	272.47	7.34	0.02	5.57	-7.13		**1**
M4	a*FD^2	**0.0475**			0.90	24.42	26241.01	24.15	-0.63	20.08	0.97	0.99	0.90	288.58	23.45	0.00	1.05			2
M1	a*FL + b	**6.8725**	**-311.718**		0.80	34.55	51343.88	33.78	0.00	27.55	0.94	0.99	0.80	320.78	55.65	0.00	7.55	-15.51		3
Reed30																				
M2	a*FD + b	**9.3255**	**-443.701**		0.94	17.99	13912.04	17.58	0.00	13.52	0.98	1.00	0.94	262.02	9.72	0.01	4.0	-6.2		**1**
M4	a*FD^2	**0.0453**			0.86	25.95	29637.53	25.66	-1.56	21.66	0.95	0.99	0.86	294.06	41.76	0.00	1.5			2
M1	a*FL + b	**7.0449**	**-325.924**		0.83	29.02	36221.43	28.37	0.00	22.30	0.95	0.99	0.83	305.08	52.79	0.00	6.8	-12.0		3
Mo=ixed																				
M7	a+ b*FL + c*FD	**-435.118**	**1.8844**	**7.1102**	0.93	20.23	90851.70	18.84	0.00	15.44	0.98	0.99	0.93	1327.16	0.00	1.00	-3.0	5.2	2.2	**1**
M2	a*FD + b	**7.7671**	**-301.595**		0.82	32.86	240785.37	29.00	0.00	27.08	0.95	0.99	0.82	1519.21	192.04	0.00	3.1	-5.8		2
M4	a*FD^2	**0.0491**			0.81	33.77	255523.93	30.18	-0.41	27.80	0.94	0.98	0.81	1535.16	208.00	0.00	0.8			3

Fig. 4. Relationship between estimated and measured total aboveground biomass of the 12 cross- validation test fruits. Circles are the volume estimates calculated using the full data set equations and the crosses are the estimates calculated using the cross-validation training dataset equations. On fig. 3, the letter E, F, H, N, R and M refers Ettinger, Fuerte, Hass, Nabal, Reed30 and Mixed cultivar data.

3.3. Conclusions and recommendation

This study provided the first avocado cultivar-specific and mixed-cultivar generalized allometric equations to estimate avocado fruit volume non-destructively. Among tested model forms, VM2 (for cultivar-specific model), and VM7 (generalized model) have passed all rigorous verification and cross-validation statistical tests, confirming that the models have sufficient skill to estimate fruit volume from easily measurable parameters. Our best allometric models (VM2 and ~~VM2~~) explained > 87% of the variation in measured fruit volumes of each cultivar. A high degree of correlation ($R^2 > 0.93$) between measured and estimated fruit volume provided quantitative evidence of the validity of the selected volume estimation models. The allometric equation developed in this research could be practical in the estimation of avocado fruit volume and applicable under field conditions. Besides, the generalized mixed-cultivar model can reliably be used to estimate avocado fruit volume when cultivar type is unknown. Our finding revealed that in the situations where fruit length and diameter measurements are possible and/or where measurements of volume would be inconvenient, or time consuming, site- and cultivar-specific allometric equations can be used to estimate fruit volume while it on the tree. Therefore, the allometric equations generated in this study could play a considerable role in improving data availability on avocado fruit physical appearance which is critical to assess the quality and taste of fresh product, which in turn, influencing the purchase decision of customers. It can also potentially assist horticulturists, agronomists, and physiologists to estimate fruit volume of avocado accurately and to carry out yield estimation prior to harvesting.

4. Acknowledgements

We thank the local community and local administration offices in the study area for their support during fieldwork. We are also grateful to Mr. Eyuel Tesfaye, and Mr. Demeke Beyen for their facilitation to the field work and organizing the team of experts for data collection. The contents of this document are solely the responsibility of the author/s and does not necessarily represent the official views of USAID or the U.S. Government or that of the institutional position of ICRAF (World Agroforestry).

5. Funding

This study was conducted with the financial support of the Africa Rising Project- a program financed by the United States Agency for International Development (USAID) as part of the United States Government's Feed the Future Initiative.

6. Authors' contributions

"Mulugeta Mokria, Aster Gebrekirstos, Hadia Said, Kiros Hadgu, Niguse Hagazi, Workneh Dubale, Achim Bräuning" designed the study. Mulugeta Mokria collected the sample fruits across the study sites, performed all sample preparation, measurements and carried out the first analysis and wrote the first version of the manuscript, which was intensively discussed and revised by all authors".

7. Conflict of interest

The authors declare no competing interests

8. Reference

1. FAOSTAT. Agricultural production data [Internet]. 2020 [cited 2020 Dec 25]. Available from: http://www.fao.org/faostat/en/#data/QC.

2. Altendorf S. Major tropical fruits market review 2018. [Internet]. Rome, FAO. The; 2019.

Available from: www.sykescottages.co.uk

3. Tadesse A. Market Chain Analysis Of Fruits For Gomma Woreda, Jimma Zone, Oromia National Regional State. Haramaya University; 2011.

4. Faris A. Review on Avocado Value Chain in Ethiopia. Ind Eng Lett. 2016;6(3):33–40.

5. Megerssa B. The role of avocado production in coffee based farming systems of South Western Ethiopia : The case of Jimma zone. African J Agric Res. 2013;8(19):2285–96.

6. Dorantes L, Parada L, Ortiz A. Post-harvest Operations. Mejía AD, editor. 2004.

7. Palou A, Serra F, Pico C. General aspects on the assessment of functional foods in the European Union. Eur J Clin Nutr. 2003;57:S12–7.

8. USDA. Avocados, Raw, all commercial varietie. [Internet]. Vol. 28, https://fdc.nal.usda.gov/fdc-app.html#/food-details/171705/nutrients. 2020. p. 1–43. Available from: doi/10.1177/1120700020921110

9. Fulgoni VL, Dreher M, Davenport AJ. Avocado consumption is associated with better diet quality and nutrient intake, and lower metabolic syndrome risk in US adults: Results from the National Health and Nutrition Examination Survey (NHANES) 2001-2008. Nutr J. 2013;12(1):1–6.

10. Shumeta Z. Avocado Production and Marketing in Southwestern Ethiopia. Trends Agric Econ. 2010;3(4):190–206.

11. Elias S. Supply Chain Analysis of Avocado and Mango Fruits in Gedeo Zone. 2016;6(9):46–55.

12. Terheggen A, Chains GV, Trade C, Livelihoods L. Ethiopia ’ s Potential Role in the

Global Avocado Market. 2019;(October).

13. Kadri Bozokalfa M, Kilic M. Mathematical Modeling in the Estimation of Pepper (Capsicum annuum L.) Fruit Volume. Chil J Agric Res. 2010;70(4):626–32.

14. Wang Z, Walsh KB, Verma B. On-tree mango fruit size estimation using RGB-D images. Sensors (Switzerland). 2017;17(12):1–15.

15. Ngouajio M, Kirk W, Goldy R. A simple model for rapid and nondestructive estimation of bell pepper fruit volume. HortScience. 2003;38(4):509–11.

16. Moreda GP, Ortiz-Cañavate J, García-Ramos FJ, Ruiz-Altisent M. Non-destructive technologies for fruit and vegetable size determination - A review. J Food Eng. 2009;92(2):119–36.

17. Riga FT. Biomass yield and nutritive value of sweet lupine in mid altitudes of Lemo District, Hadiya Zone, Southern Ethiopia. [Internet]. Vol. 9. Wolaita Sodo University; 2017. Available from: https://doi.org/10.1016/j.tmaid.2020.101607%0Ahttps://doi.org/10.1016/j.ijsu.2020.02.034%0Ahttps://onlinelibrary.wiley.com/doi/abs/10.1111/cjag.12228%0Ahttps://doi.org/10.1016/j.ssci.2020.104773%0Ahttps://doi.org/10.1016/j.jinf.2020.04.011%0Ahttps://doi.o

18. CSA-Ethiopia. Summary and Statistical Report pf the 2007 Population and Housing Census [Internet]. Addis Ababa, Ethiopia; 2008. Available from: http://www.ghbook.ir/index.php?name=مجموعه مقالات دومین همایش اندیشه سراسری رسانه و تلویزیون سکولاریسم&option=com_dbook&task=readonline&book_id=13629&page=108&chkhashk=03C706812F&Itemid=218&lang=fa&tmpl=component

19. Mekonnen K, Yasabu S, Gebremedhin B, Woldemeskel E, Tegegne A, Thorne P, editors. Promoting productivity and market access technologies and approaches to improve farm income and livelihoods in Ethiopia: Lessons from action research projects. Proceedings of a Workshop, ILRI, Addis Ababa, 8–9 December 2016. Nairobi, Kenya: ILRI. In Nairobi, Kenya: ILRI,; 2019. Available from: http://africa-rising.

20. Schaffer B, Baranowski RM. Sample size estimates for avocado yield experiments. J Am Soc Hortic Sci. 1986;111(6):985–7.

21. Zilkah S, Klein I. Growth Kinetics and Deremiantion of Shape and Size of Small and Large Avocado Fruits Cultivar' Hass' On the Tree. Sci Hortic (Amsterdam). 1987;32(195):195–202.

22. Marini RP. Estimating mean fruit weight and mean fruit value for apple trees: Comparison of two sampling methods with the true mean. J Am Soc Hortic Sci. 2001;126(4):503–10.

23. Marini RP, Schupp JR, Baugher TA, Crassweller R. Sampling apple trees to accurately estimate mean fruit weight and fruit size distribution. HortScience. 2019;54(6):1017–22.

24. Erickson LC, Kikuta Y. Determination of Surface Area and Volume of Avocado Fruits. 1965;(Figure 1).

25. Sileshi GW. A critical review of forest biomass estimation models, common mistakes and corrective measures. For Ecol Manage [Internet]. 2014;329:237–54. Available from: http://dx.doi.org/10.1016/j.foreco.2014.06.026

26. Gill JL. Outliers, residuals, and influence in multiple regression. J Anim Breed Genet. 1986;

27. Mahanti KK, Rathan SK, Ravanappa V, Vadivel S, Ramakrishnaiah JB. A Regression Model for Nondestructive Fruit Volume Estimation in Karond a (C Arissa C Arandus L Estimation Carandus L .). Suppl Agron. 2016;11(1):659–62.

28. Johnston R, Jones K, Manley D. Confounding and collinearity in regression analysis: a cautionary tale and an alternative procedure, illustrated by studies of British voting behaviour. Qual Quant. 2018;52(4):1957–76.

29. Seong C, Herand Y, Benham BL. Automatic calibration tool for hydrologic simulation program-FORTRAN using a shuffled complex evolution algorithm. Water (Switzerland). 2015;7(2):503–27.

30. Mokria M, Gebrekirstos A, Abiyu A, Noordwijka M Van, Braüning A. Multi-century tree-ring precipitation record reveals increasing frequency of extreme dry events in the upper Blue Nile River catchment. Glob Chang Biol. 2017;23:2222–49.

31. Mokria M, Mekuria W, Gebrekirstos A, Aynekulu E, Belay B, Gashaw T, et al. Mixed-species allometric equations and estimation of aboveground biomass and carbon stocks in restoring degraded landscape in northern Ethiopia. Environmeantal Res Lett. 2018;00:00.

32. Rahman SMAK, Sathik MM, Kannan KS. Multiple Linear Regression Models in Outlier Detection. Int J Res Comput Sci. 2012;2(2):23–8.

33. Negash M, Starr M, Kanninen M, Berhe L. Allometric equations for estimating aboveground biomass of Coffea arabica L. grown in the Rift Valley escarpment of Ethiopia. Agrofor Syst [Internet]. 2013 Apr 4 [cited 2013 Dec 3];87(4):953–66. Available from: http://link.springer.com/10.1007/s10457-013-9611-3

34. Burnham KP, Anderson DR. Model Selection and Multimodel Inference: A Practical Information-Theoretic Approach (2nd ed) [Internet]. Vol. 172, Ecological Modelling. 2002. 488 p. ~~Available from: http://linkinghub.elsevier.com/retrieve/pii/S0304380003004526~~

35. Bayram S, Alper Arslan M, Turgutoğlu E, Erkan M. The performance of some avocado cultivars under Mediterranean coastal conditions in Turkey. J Food, Agric Environ. 2012;10(2):588–92.

36. Bayram S, Tepe S. Determination of some physicochemical properties in fruits of some avocado (Persea americana Mill.) cultivars during the harvesting periods. Derim. 2019;(May).

37. Janice DA, John A, Jemmy FT. Morphological characteristics of avocado (Persea americana Mill.) in Ghana. African J Plant Sci. 2018;12(4):88–97.

38. Lee SK, Young RE, Schiffman PM, Coggins CW. Maturity Studies of Avocado Fruit Based on Picking Dates and Dry Weight. J Amer Soc Hort Sci. 1983;108(3):390–4.

39. Woolf A, Clark C, Terander E, Phetsomphou V, Hofshi R, Arpaia L, et al. Measuring avocado maturity; ongoing developments Allan. THE ORCHARDIST. 2003.

40. Barmore CR. Avocado Fruit Maturity. Proc First Int Trop Fruit Short Course Avocado JW Sauls, RL Phillips LK Jackson (eds) Gainesv Fruit Crop Dept, Florida Coop Ext Serv Inst Food Agric Sci Univers. 1976;103–9.

41. Ammann CM, Wahl ER. The importance of the geophysical context in statistical evaluations of climate reconstruction procedures. Clim Change. 2007;85(1–2):71–88.

42. Krause P, Boyle DP, Base F. Comparison of different efficiency criteria for hydrological model assessment. Adv Geosci [Internet]. 2005;5(89):89–97. Available from: http://www.adv-geosci.net/5/89/2005/

43. Moriasi DN, Arnold JG, Liew MW Van, Bingner RL, Harmel RD, Veith TL. Model evaluation guidelines for systematic quantification of accuracy in watershed simulations. Am Soc Agric Biol Eng. 2007;50(3):885–900.

44. Vanderlei RS, Silva JV, Moura F de BP. Biometry and non-destructive allometric model for estimating Babassu (Attalea speciosa) fruit volume and densities. Rev Ouricuri. 2019;9(2):1–10.

45. Kejun Y, Ruihong S. An Introduction to the New Estimation Formulae of Apple Fruit Volume and Surface Area. 2013.

Figure
Click here to access/download;Figure;Fig. 1.tiff

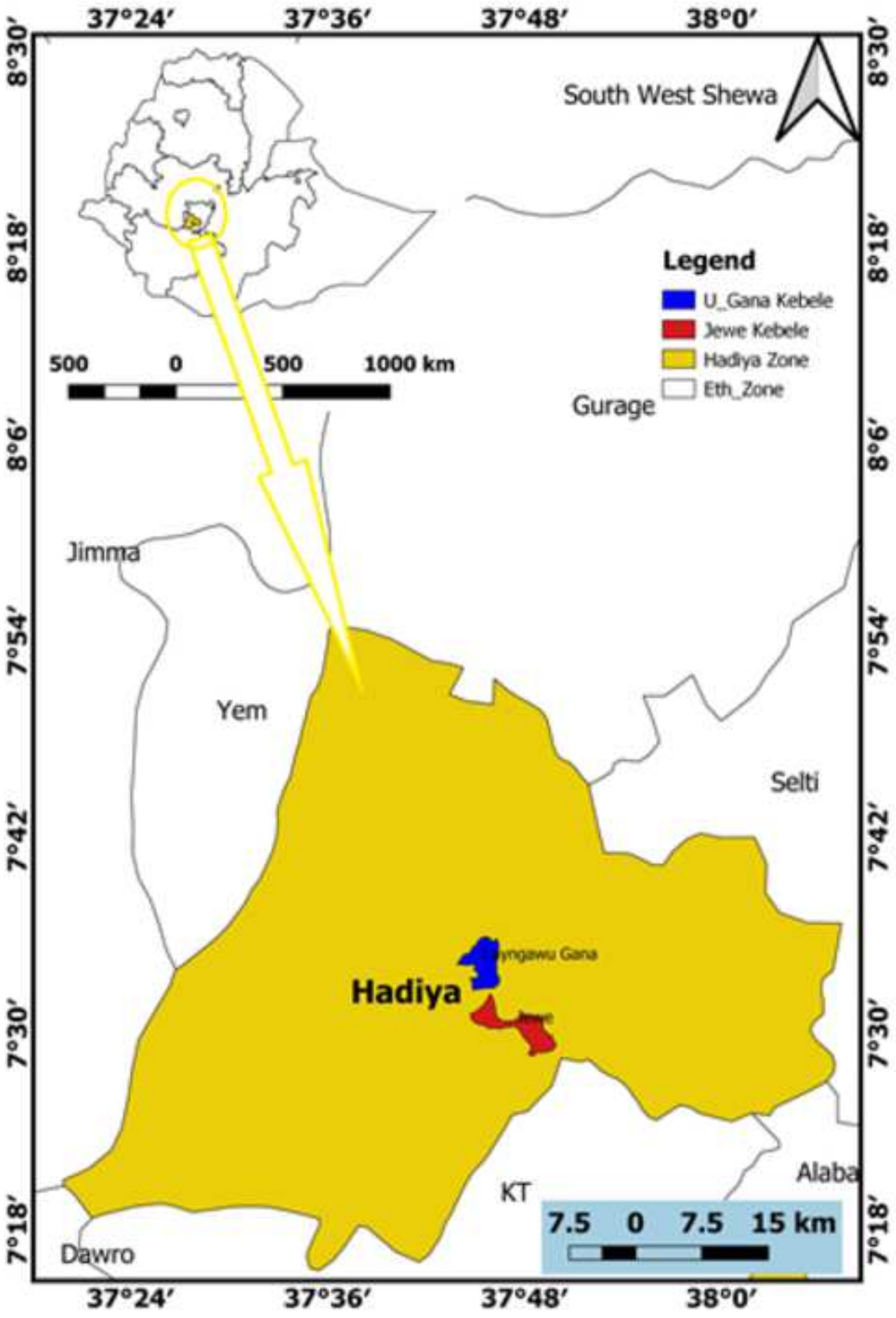

Figure
Click here to access/download;Figure;Fig. 2.TIF

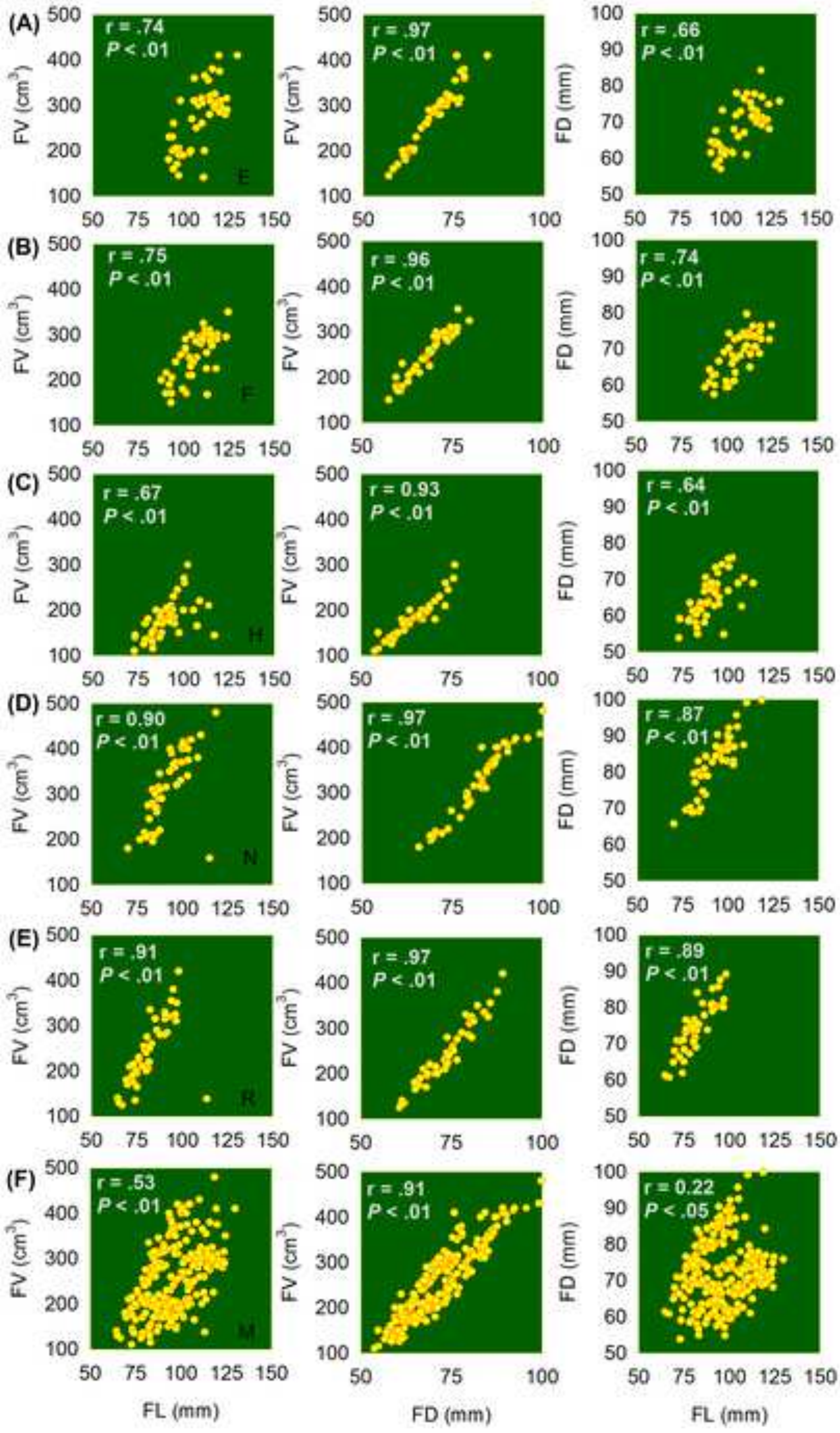

Figure
Click here to access/download;Figure;Fig. 3.TIF

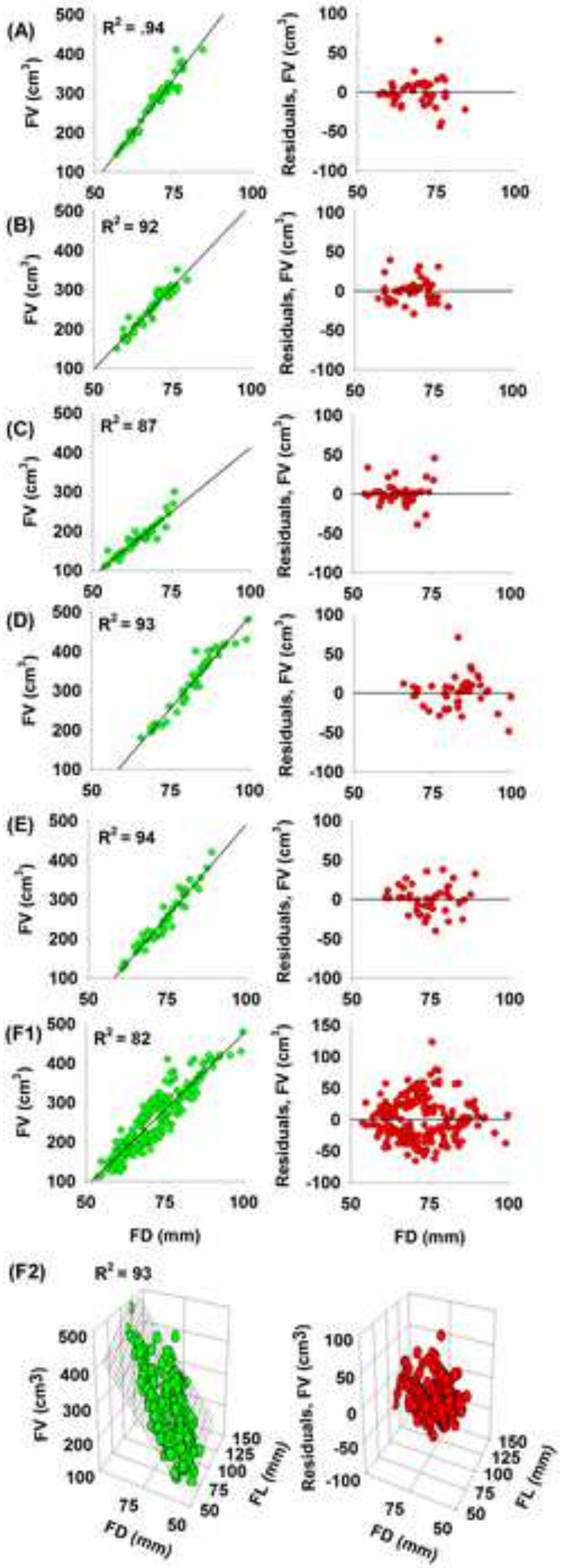

Click here to access/download;Figure;Fig. 4.TIF

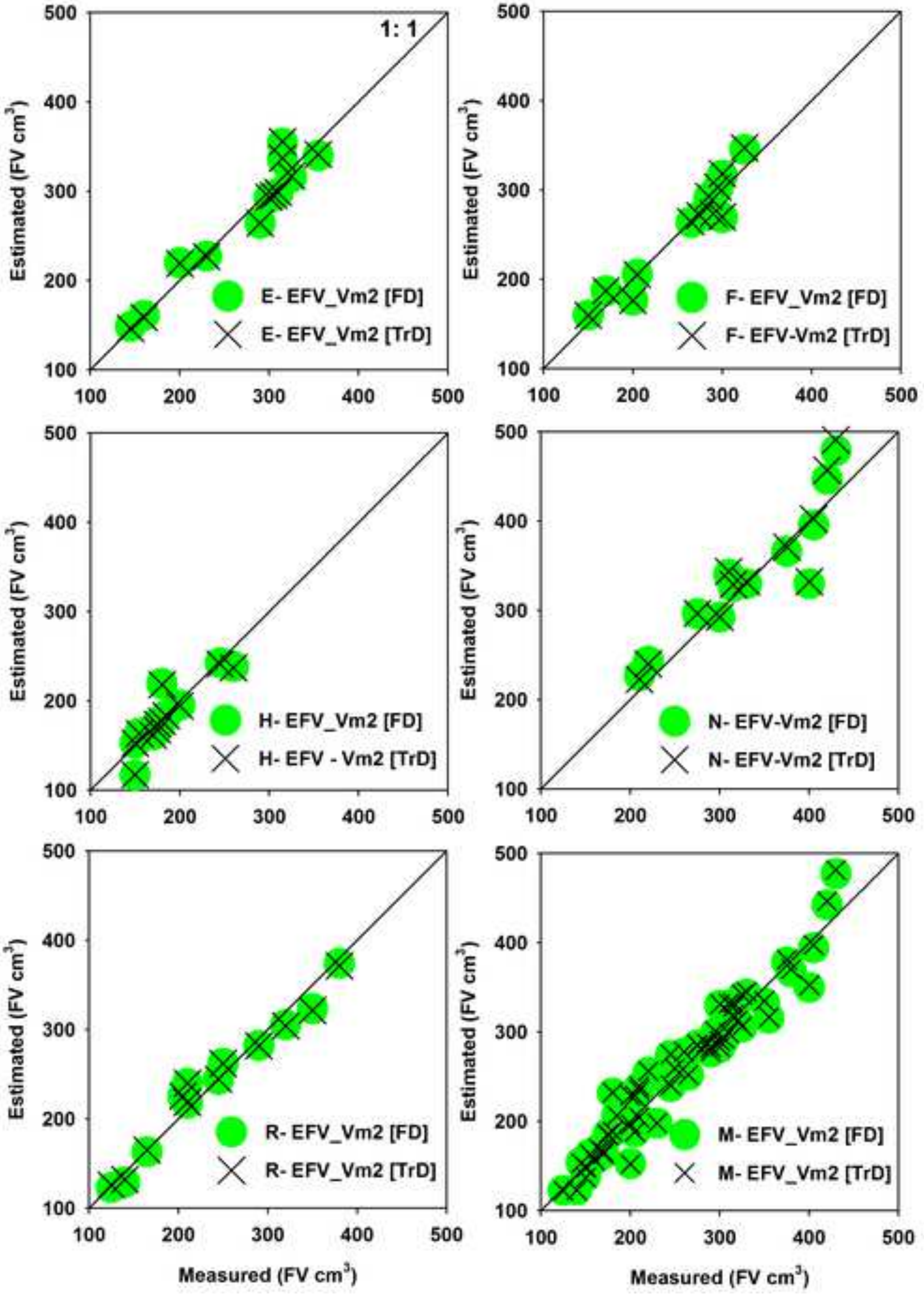

Supplement: Attachment — Submitted filename: PONE-D-21-36255_reviewer.pdf [file pone.0263564.s003.pdf]
