# Supplementary material for: Volume estimation models for avocado fruit
Source: PLoS One. 2022 Feb 3;17(2):e0263564. doi: 10.1371/journal.pone.0263564 (PMC8812970; doi:10.1371/journal.pone.0263564)
Supplement: Attachment — Submitted filename: Response to Reviewers.docx [file pone.0263564.s004.docx]

# **Response to reviewers comment**

## **ID: PONE-D-21-36255**

## **Title: Volume estimation models for tropical fruit**

## **Dear Editor**

First, we would like to express our sincere appreciation to you and the anonymous reviewers for their very insightful and constructive comments and suggestions to our manuscript. Kindly find the response to each of the comments below. We believe we were able to address all suggestions and comments adequately, otherwise please contact us.

**Response to the editor:** *We are very great full for your comment and suggestion. We amended the structure/style of the revised manuscript as the journal guideline. Regarding, fig 1, we reproduced the map by taking the shapefiles from openAFRICA ( open sources Africa Shapefiles, please see: -*[*https://open.africa/dataset/africa-shapefiles*](https://open.africa/dataset/africa-shapefiles)*);* [*http://geoportal.icpac.net/layers/geonode%3Aafr_g2014_2013_0*](http://geoportal.icpac.net/layers/geonode%3Aafr_g2014_2013_0)*.*

On behalf of all co-authors,

Sincerely,

**Mulugeta Mokria**

# **Response to reviewer #1**

This study provided the first avocado cultivar-specific and mixed-cultivar generalized allometric equations to estimate avocado fruit volume non-destructively.

**Major comments:**

**Comment #1.** The title of the article does not provide a clear picture of the subject under consideration. The title implies that a general allometric model for all tropical crops is provided, but this model was only used for non-destructive measurement of avocado fruit volume. As a result, it is suggested that the article be given a more accurate title.

**Response #1**: We thank the reviewer for making a very important point and we revised the title to “Volume estimation models for avocado fruit” *please see line 1 in the revised manuscript.*

**Comment #2.** Avocado is a highly variable species and three botanical subspecies or ecological races of P. americana Mill. were recognized: Mexican (M) (var. drymifolia), Guatemalan (G) (var. guatemalensis), and West Indian (WI) (Antillean) (var. americana). Cultivars derived from Guatemalan and Mexican races and their hybrids are grown primarily in subtropical climates and have physiological adaptations to cooler temperatures, as opposed to cultivars derived from West Indian race or WI­hybrids, which are adapted to tropical climates.

Regarding this horticultural fact, there must be consistency throughout the manuscript's text as well as title. Now, none of the cultivars studied in this study are of tropical origin.
Using the words 'tropical' in the title and line 17 and 'subtropical' in line 40 made inconsistencies that must be addressed.

**Response #2:** The reviewer makes a very important point, and we are grateful for that. Thus, we amended the titles and avoided the inconsistency in using the term “Tropical” through the manuscripts. *Please see lines 1, 17, 38 in the revised manuscript.*

**Comment #**3. According to line 117: “model performance was checked using various goodness-of-fit statistics, such as the Coefficient of Determination (R2), Standard Error of Estimate (SEE), Index of Agreement (D), Mean Absolute Bias (MAB), Percent Bias (PBIAS), Root Mean Square Error (RMSE), Prediction Residuals Sum of Squares (PRESS), Reduction of error (RE), and Coefficient efficiency (CE)”.

As is obvious, the regression was run separately for the actual fruit volume and each of the independent variables, such as fruit length, fruit weight, and fruit diameter. In other words, a single factor regression has been investigated (Length with volume, diameter with volume and weight with volume). As you are aware, mathematical relationships such as regression between the sum of length and diameter with volume, regression between the multiplication of length and diameter by volume, or regression between the square of length + diameter by volume are preferable. It is strongly advised to provide better estimation by providing more mathematical relationships between fruit length and diameter and regressing them with fruit volume so that a better choice between them can be made.

**Response #3:** The reviewer makes a very important point, and we are grateful for that. As hinted by the reviewer, we have tested about 9 model forms (*see the supplementary information - S2_Model Performance statistics.xls*) and we selected the three most potential models and their estimation efficiency also depends on the varieties. *Please see the revised manuscript.*

**Comment #**4. According to section 2.3: “Cultivar-specific and mixed-cultivar generalized avocado fruit volume (FV) estimation models were developed using linear and non-linear regression equations based on either fruit diameter, fruit length alone or both fruit length and diameter at the same time as independent variables”.

Please include both linear and non-linear regression equations in the table, as well as the Goodness of Fit for the linear equation. If a non-linear equation is used, please explain it using terms such as logarithm, polynomial, and so on. Non-linear regression can provide more accurate predictions and is especially valuable for biological data. The value and strength of the proposed model are increased by including these items.

**Response #4:** We thank for the suggestions. **We provided b**oth linear and non-linear regression equations and with their goodness of fit statistics (***Please see the Supplementary information-*** *S2_Model Performance statistics.xls*). However, for simplicity, we provided only the three most performed equations in the main text and supplied the statistical performances of all tested models in the supplementary information document with more explanation (*S2_Model Performance statistics.xls)*.

**Comment #**5. According to line 128: “To validate the best fitting equation for volume estimation, model cross-validation was conducted following a split-sample approach in which 45 measured fruit sample were partitioned into two sets, 33 for ‘‘training’’ (i. e., to develop the equations) and the remaining 12 fruit samples for ‘‘testing’’ the equations”.

Twelve fruits from each cultivar were used for testing (validation), which appears insufficient and represents a small statistical population. Moreover, validation requires sampling from other gardens or areas in the same climate. In other words, performing large-scale sampling will result in strong validation with a high correlation coefficient.

**Response #5:** The reviewer makes a very important point. We share the concern of the reviewers’ and agree on model validation using large and independent data will result greater confidence on model selection and use. However, we could not find such independent data in Ethiopia, and we also collected only 45 sample fruit from each variety. There for, we indicated as the limitation of the manuscript and suggested the need for further study using large database (if available). *Please see lines 250 -252, in the revised manuscript*.

**Comment #**6. After obtaining the model and estimating the data, regression validation should be performed to determine whether a suitable correlation coefficient exists. Please include graphs of validation-related correlations.

**Response #6:** We thanks the review for pointing out this and we have provided a cross-validation graph. *please see line 225, Fig 5, in the revised manuscript.*

Minor comments:
**Comment #**1. Line 17: Mill -> Mill (non-italicized)

**Response #1:** *We revised the text accordingly, please see line 17 in the revised manuscript.*
**Comment #**2. Line 17: remove "genus Persea" at the end.

**Response #2:** We removed the word “genus Persea, *please see line 17”in the revised manuscript.*
Comment #3. Line 19: The physical characteristic -> The physical characteristics

**Response #3:** *Thanks, we revised the text accordingly, please see line 18 in the revised manuscript.*
**Comment #**4. Line 23: "five wildly distributed avocado verities" -> Q: five varieties or cultivars? As mentioned in the materials and methods section, they are 5 avocado cultivars.

**Response #4:** We thanks the review for pointing out this and changed the text *to “cultivars” through the manuscript, please see line 23 in the revised manuscript.*
**Comment #**5. Line 23: "five wildly distributed avocado verities" -> … varieties

**Response #5:** *Thanks, corrected accordingly, please see line 23 in the revised manuscript*
**Comment #**6. Line 24: found between Fruit diameter -> fruit

**Response #6:** *We revised the text accordingly, please see line 23 in the revised manuscript***.**
**Comment #**7. Line 58: small scall avocado farming -> scale

**Response #7:** *Thanks, corrected accordingly, please see line 55 in the revised manuscript.*
**Comment #**8. Line 59: information of fruit size are critical factors -> is critical

**Response #8:** *Corrected, please line 56 in the revised manuscript*  **Comment #**9. Line 73: five avocado verities -> … varieties (Q: five varieties or cultivars?)

**Response #9:** *Thanks, changed to cultivars through the manuscript, please see line 70 in the revised manuscript.*  **Comment #**10. Line 77: Materials and methods -> Materials and Methods

**Response #10:** *We revised the text accordingly, please see line 74 in the revised manuscript***.**
**Comment #**11. Line 78: 2.1 -> 2.1.

**Response #11:** *Thanks, revised accordingly, please see line 75 in the revised manuscript.*
**Comment #**12. Line 101: classified in to three size -> into

**Response #12:** *We revised the text accordingly, please see line 101 in the revised manuscript*

**Comment #**13. Line 130: i. e. -> i.e.

**Response #13:** *Thanks, corrected accordingly***,** *please see line 133 in the revised manuscript*

**Comment #**14. Line 139: Fruit volume -> fruit volume

**Response #14:** *We revised the text accordingly, please see line 140 in the revised manuscript*
**Comment #**15. Line 145: Result and discussion -> Results and Discussion

**Response #15:** *We revised the text accordingly, please see line 147 in the revised manuscript*
**Comment #**16. Line 234: (VM2 and VM2) -> ?

**Response #16:** *Corrected and VM2 is changed to VM7, please see line 235 in the revised manuscript.*
**Comment #**17. Line 312: reference # 18: ?

**Response #17:** *We thank the reviewer for pointing out this, and corrected it accordingly, please lines 314-15, in the revised manuscript.*

**Other comments:**

**Comment #**1. The main body and reference section must be adjusted in accordance with the author's guidelines.

**Response #1:** *We thank the reviewer for pointing out this. We corrected the main body and the reference style as per the journal user guide.*

**Comment #**2. The manuscript must be significantly improved and rewritten as a result of comments and suggestions. All of the issues raised should be addressed. The manuscript must then be reassessed for quality and suitability for publication in PLOS ONE.

**Response #2:** *We thank the review for constructive comments and suggestions to our manuscript. We believe we were able to include all suggestions and comments in an adequate way, otherwise please contact us.*

# **Response to reviewer #2**

**Reviewer #2:**

Manuscript presents allometric models to non-destructively predict avocado fruit volume under both cultivar-specific and mixed-cultivar production systems. Introduction, Materials and Methods, and Results and Discussion sections are adequately written and justify the claims made in the manuscript. Data collection method, and statistical tests sufficiently verify the models. I suggest to maturity level of fruit at the time of harvest and add a colored photograph showing morphological differences among avocado fruits from studied cultivars. Since, all models have some limitations, it would be appropriate to add limitations for these models, too. Some English language improvements are suggested in the reviewed version of the manuscript.

**Response #:** *We thank the review for constructive comments and suggestions to our manuscript. We added a colored photograph showing morphological differences of each avocado cultivars (Please see Fig 1 lines 106-107, and S1_Colored photograph of five Avocado cultivars.pdf, line 394 in the revised manuscript. In addition, we have added a recommendation to conduct a similar study using a large dataset to improve model performances and that can be used across regions (Please lines 249- 252, in the revised manuscript). We believe we were able to include all suggestions and comments including some language aspects in an adequate way.*
