# Supplementary material for: Volume estimation models for avocado fruit
Source: PLoS One. 2022 Feb 3;17(2):e0263564. doi: 10.1371/journal.pone.0263564 (PMC8812970; doi:10.1371/journal.pone.0263564)
Supplement: Attachment — Submitted filename: Response to Reviewers.docx [file pone.0263564.s005.docx]

# **Response to reviewers comment**

## **ID: D-21-36255R1**

## **Title: Volume estimation models for avocado fruit**

## **Dear Editor**

First, we would like to express our sincere appreciation to you and the anonymous reviewers for their time in reading the revised manuscript and providing constructive comments and suggestions which is relevant in improving the manuscript. Kindly find the response to each of the comments below. We believe we were able to address all suggestions and comments adequately, otherwise please contact us.

On behalf of all co-authors,

Sincerely,

**Mulugeta Mokria**

**Response to Editor Comments**

**Comment #1:** Please review your reference list to ensure that it is complete and correct. If you have cited papers that have been retracted, please include the rationale for doing so in the manuscript text, or remove these references and replace them with relevant current references. Any changes to the reference list should be mentioned in the rebuttal letter that accompanies your revised manuscript. If you need to cite a retracted article, indicate the article’s retracted status in the References list and also include a citation and full reference for the retraction notice.

**Response #1:** We are very grateful for your comment and suggestion. We checked the lists of the reference and they are complete and correct, *please see lines 279-405 in the revised manuscript*. In addition, in response to the reviewer's suggestion, we have cited a few more articles and changes are indicated in the revised manuscript. *Please see lines, 39, 97, 279-283, 330, 403, in the revised manuscript.*

# **Response to reviewer #1**

**General comment.** Thank you for your efforts in revising the manuscript in an appropriate manner. I believe that the revised manuscript has significant improvements. In other words, you answered all of the questions and made all of the necessary changes. In addition, the revised manuscript now includes significant and acceptable English language improvements, as well as a more in-depth discussion with adequate presentation and interpretation for each of the research findings.

**General response:** We are also grateful for your time in reading the revised manuscript and providing constructive comments and suggestions that considerably helped us to improve the manuscript.

**Comment #1:** Note that, regarding your responses #3 and #4 to reviewer #1, you must state your arguments in the main text of the manuscript as declarative sentences in order to inform readers. As a result, readers may be able to better understand the details of the reports and findings.

**Response #1:** The reviewer makes a very important point, and we are grateful for that. As indicated by the reviewer, we added a statement that will guide readers to better understand the details of the type of model tested, and their performance statices, as well as the report in general*. Please see lines 186-191, in the revised manuscript.*

# **Response to reviewer #2**

**General comment:** Manuscript has been thoroughly reviewed. After review, the manuscript is in much better shape. Authors have properly incorporated or justified all suggested improvements.

**General response:** Thanks a lot and we are also grateful for your time in reading the revised manuscript and positive feedbacks.

**Response to Academic Editor Comments**

**Comment #1:** There are two Hass cultivars in the figure 2 whereas in tables the names are not same. Please be uniform in the whole manuscript about the names of the avocado cultivars. In addition, it would be better if the authors provide race information for the each cultivar. For your convenience, please refer to this publication i.e. https://doi.org/10.1016/j.scienta.2019.109008

**Response #1:** Thanks a lot for your critical comments and suggestions to read an important article. In Fig 2, both are the same Hass cultivar. But, now we removed the replicated photos of the same cultivar to avoid further confusion and consistently used the same cultivar's name. *Please see the revised Fig2 and the revised manuscript*. Moreover, we further referred to the suggested article and provided “race” information for each cultivar. *Please see lines 95-97 (in the main body) and lines 330-332 (in the reference list).*
